# *Nf1* Loss and Ras Hyperactivation in Oligodendrocytes Induce NOS-Driven Defects in Myelin and Vasculature

**DOI:** 10.1016/j.celrep.2013.08.011

**Published:** 2013-09-12

**Authors:** Debra A. Mayes, Tilat A. Rizvi, Haley Titus-Mitchell, Rachel Oberst, Georgianne M. Ciraolo, Charles V. Vorhees, Andrew P. Robinson, Stephen D. Miller, Jose A. Cancelas, Anat O. Stemmer-Rachamimov, Nancy Ratner

**Affiliations:** 1 Division of Experimental Hematology and Cancer Biology, Cincinnati Children’s Hospital Medical Center, University of Cincinnati College of Medicine, Cincinnati, OH 45229, USA; 2 Division of Pathology, Cincinnati Children’s Hospital Medical Center, University of Cincinnati College of Medicine, Cincinnati, OH 45229, USA; 3 Division of Neurology, Cincinnati Children’s Hospital Medical Center, University of Cincinnati College of Medicine, Cincinnati, OH 45229, USA; 4 Department of Microbiology-Immunology, Feinberg School of Medicine, Northwestern University, Chicago, IL 60611, USA; 5 Department of Pathology, Massachusetts General Hospital and Harvard Medical School, Boston, MA 02114, USA

## Abstract

Patients with neurofibromatosis type 1 (NF1) and Costello syndrome Rasopathy have behavioral deficits. In NF1 patients, these may correlate with white matter enlargement and aberrant myelin. To model these features, we induced *Nf1* loss or HRas hyperactivation in mouse oligodendrocytes. Enlarged brain white matter tracts correlated with myelin decompaction, downregulation of claudin-11, and mislocalization of connexin-32. Surprisingly, non-cell-autonomous defects in perivascular astrocytes and the blood-brain barrier (BBB) developed, implicating a soluble mediator. Nitric oxide (NO) can disrupt tight junctions and gap junctions, and NO and NO synthases (NOS1–NOS3) were upregulated in mutant white matter. Treating mice with the NOS inhibitor NG-nitro-L-arginine methyl ester or the antioxidant N-acetyl cysteine corrected cellular phenotypes. *CNP-HRasG12V* mice also displayed locomotor hyperactivity, which could be rescued by antioxidant treatment. We conclude that Nf1/Ras regulates oligodendrocyte NOS and that dysregulated NO signaling in oligodendrocytes can alter the surrounding vasculature. The data suggest that anti-oxidants may improve some behavioral deficits in Rasopathy patients.

## INTRODUCTION

Oligodendrocytes are the myelin-forming glial cells of the CNS. Changes in myelination have recently been implicated in CNS sensory and motor control, and in cognition (Zatorre et al., 2012). However, the role of oligodendrocytes and myelin in genetic diseases in which myelination changes have been implicated and cognitive deficits exist, as in Rasopathies such as neurofibromatosis type 1 (NF1) and Costello syndrome, has not been studied.

Rasopathies are inherited disorders in which individuals have mutations in Ras signaling pathway genes. Activating mutations in Ras proteins themselves, as in Costello syndrome (caused by mutations in *H-Ras*), or inactivation of Ras-GAPs, as in NF1 patients (caused by mutations in the *NF1* gene), result in aberrant signaling to Ras effectors (Gysin et al., 2011). Low-grade pilocytic astrocytomas arise in white matter tracts in about 20% of NF1 patients (Williams et al., 2009). Results from diffusion tensor MRI (DTI) suggest that global changes occur in the brains of NF1 patients (van Engelen et al., 2008; Karlsgodt et al., 2012). T2 hyperintense regions in NF1 patients’ brains correlate with more profound DTI changes. On pathological analysis, these T2 hyperintense spots may be regions of dysmyelination (DiPaolo et al., 1995) and in some studies have been correlated with behavioral changes in NF1 patients (Hyman et al., 2007). More than half of NF1 patients present with CNS defects, learning and memory deficits, and/or hyperactivity (Acosta et al., 2012). Although the cellular changes that cause these deficits remain to be determined, more than half of NF1 patients are affected by macrocephaly, and increased size of the corpus callosum is associated with behavioral deficits (Pride et al., 2010). NF1 protein is highly expressed in rodent and human oligodendrocytes (Daston et al., 1992); therefore, we speculated that *NF1* loss in oligodendrocytes could play a role in both the pathology and physiology of brain dysfunction in NF1 patients. The brain histology in Costello syndrome has not been studied, but macrocephaly followed by microcephaly, seizures, and cognitive problems has been reported, as has dysmyelination of optic nerves and basal ganglia based on brain imaging (Tidyman and Rauen, 2008). Costello mutations include the severe *HRasG12V* mutation, which we modeled.

In this study, we examined the effects of *Nf1* loss and hyperactivation of HRas (*HRasG12V*) on oligodendrocytes in the adult brain. We identified a Ras-nitric oxide (NO) pathway that mediates altered myelin structure. Several lines of evidence have suggested key roles for reactive oxygen and nitrogen as upstream regulators and downstream effectors of Ras signaling (Ferro et al., 2012). Ras signaling through the downstream effectors MEK/ERK induces the generation of reactive oxygen species (ROS) (Heimfarth et al., 2013). Furthermore, MEK has been shown to play a critical role in oligodendrocyte formation (Li et al., 2012), and developmental *Nf1* loss throughout the brain resulted in corpus callosum enlargement that could be reduced in size by MEK inhibition (Wang et al., 2012). However, in *Drosophila*, *dNf1* loss caused a cyclic AMP (cAMP)-induced, Ras-independent upregulation of ROS (Tong et al., 2007). How loss of *Nf1* affects oligodendrocyte viability and/or function remains unknown.

Oligodendrocytes manufacture myelin. Compact myelin is a multilamellar membrane whose structural integrity depends upon identified proteins and lipids. In addition to the major myelin proteins, such as proteolipid protein (Plp), myelin contains gap junction (GJ) and tight junction (TJ) proteins. Oligodendrocyte-specific protein (OSP; claudin-11) is a TJ protein. Claudin-11 mutants showed myelin decompaction in concert with *Plp* mutation (Chow et al., 2005). Genetic loss of the oligodendrocyte connexin protein Cx32 in mice results in myelin vacuolization (Magnotti et al., 2011). Claudins and connexins can be regulated by MEK/ERK or NO (Lee et al., 2009; Radosinska et al., 2011; Beutheu et al., 2012); however, such regulation has not been studied in intact brains.

We find that cell-intrinsic Ras upregulation in oligodendrocytes deregulates the expression and/or localization of TJ and GJ proteins in the myelin sheath, and, unexpectedly, in astrocyte and endothelial cells, resulting in altered permeability of the blood-brain barrier (BBB). Mechanistically, cellular phenotypes were caused by Ras-driven upregulation of NO synthase (NOS) expression and NO production. Behavioral changes also resulted from altered Ras/NO signaling in oligodendrocytes.

## RESULTS

### *PlpCre;ERT* (PlpCre) and *CNP-HRasG12V* Model Systems Target Mature Oligodendrocytes

To target *Nf1* loss to oligodendrocytes, we used a tamoxifen-inducible PLP driver (Doerflinger et al., 2003). Plp is mainly expressed in adult white matter oligodendrocytes, but cellular targets of this driver have not been completely defined. We analyzed *PlpCre;CMV-βactin-loxP-EGFP* flanked (CAT) mice 1 day after they were dosed with tamoxifen for 3 days. The schematic in Figure S1 shows the oligodendrocyte lineage. As expected, enhanced green fluorescent protein (EGFP)+ cell labeling was enriched in white matter. We analyzed the optic nerve and corpus callosum in detail. Twenty percent of optic-nerve cells were EGFP+ and thus were affected by Cre recombinase. All EGFP+ cells in the optic nerve were double labeled with CC1 (Figure S1A), and 35% of the CC1+ population was double labeled with EGFP. In the corpus callosum, 75%–80% of EGFP+ cells were CC1+ (Figure S1A), likely indicating variable expression of CC1 in mature oligodendrocytes (Zeger et al., 2007). EGFP+ cells in white matter were negative for the progenitor marker NG2 (Figure S1B). Most EGFP+ cells had olig2+ nuclei (Figure S1C) and were CC1+ (Figure S1D). Cells expressing the astrocyte marker glial fibrillary acidic protein (GFAP; Figure S1E), the microglial marker Iba1 (Figure S1F), and the endothelial cell marker tomato lectin (Figure S1G) were EGFP−. No axons in the optic nerve or corpus callosum were EGFP+. Scattered neurons in the granule cell layers of the hippocampus and cerebellum were EGFP+, as were rare interneurons of the olfactory bulb (not shown).

The cellularity of optic-nerve cross-sections in hematoxylin and eosin (H&E)-stained paraffin sections did not change, and there was no increase in cell proliferation as assessed by bromo-deoxyuridine (BrdU) or Ki67/EGFP+, or cell death as assessed by TUNEL/caspase-3 immunohistochemistry (not shown). Furthermore, cell counts for NG2 and CC1 did not change in the optic nerve or corpus callosum (Figures S1H and S1I). Olig2 cell counts did increase in the corpus callosum, as expected from previous studies (Wang et al., 2012).

To mimic a severe form of Costello syndrome, we used a hemagglutinin (HA)-tagged *CNP-HRasG12V* mouse model (*CNP-HRas*). In this mouse, Ras-GTP is constitutively expressed (Figure S2A) as predicted for this G12V allele (Gysin et al., 2011; Patel et al., 2012). Immunohistochemical analysis confirmed HA staining primarily in the white matter of CNP-HRas animals (Figure S2C), which was absent in wild-type (WT) brain (Figure S2B). In the corpus callosum, the HA tag localized primarily to cell processes (Figure S2D). Verifying HA-Ras expression in mature oligodendrocytes, MAG+ ad-axonal membranes showed extensive overlap with the HA tag (Figure S2E) and MBP+ compact myelin was partly colocalized (Figure S2F). Astrocytes did not express *HRasG12V* (Figure S2G).

### *Nf1* Loss or HRas Activation in Oligodendrocytes Causes Optic-Nerve Enlargement, Myelin Decompaction, Loss of Claudin-11, and Altered Connexin 32 Localization

**Figure 1:**
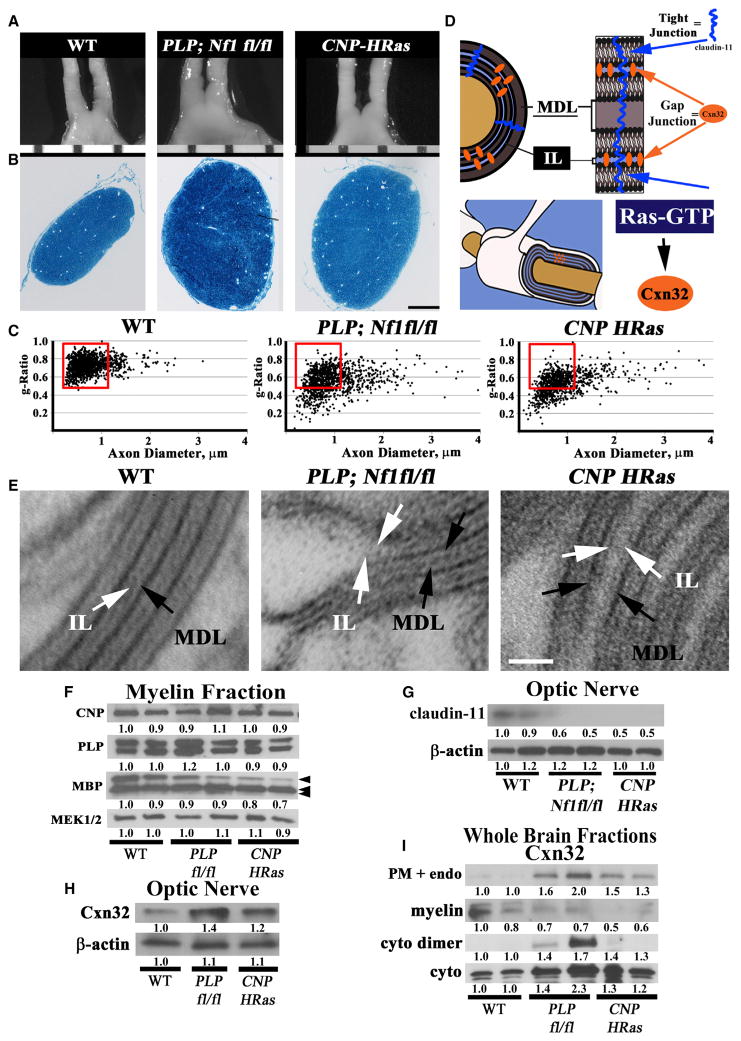
*Nf1* Loss or HRas Activation in Oligodendrocytes Causes Optic-Nerve Enlargement, Myelin Decompaction, Loss of Claudin-11, and Altered Cx32 Localization (A) Gross micrographs of optic nerves at the level of the chiasm in 12-month-old animals. Ruler shows 1 mm markings. (B) Toluidine-blue-stained semithin optic-nerve cross-sections, 1 mm rostral to the chiasm. (C) Optic-nerve g-ratio scatterplots (~5,000 axons/graph; measurements 1 mm rostral to the chiasm in three to five electron micrographs at 10,000×). Red box: 99% of WT g-ratios superimposed upon graphs of other genotypes. ANOVA, Tukey post hoc, p = 2.2 × 10^−16^. (D) Diagram of myelin compaction. Brown circle: axon cross-section; gray: major dense lines (MDLs); orange: Cx32 GJs between compact myelin layers; blue lines: claudin-11+ TJs in compact myelin. (E) Electron micrographs (scale bar, 10 nm) of WT, *PLPCre;Nf1 fl*/*fl*, and *CNP-HRas* optic-nerve myelin. White arrows: intraperiod lines (ILs); black arrows: MDLs. (F–I) Western blots of total optic-nerve lysate or whole-brain cytosolic, endosomal, or myelin fractions showing myelin proteins (F), claudin-11 (G), or Cx32 cellular localization (H and I). n = 3–5 animals/genotype/experiment. See also Figures S1, S2, S3, and S4.

To test whether white matter structure is altered by *Nf1* loss or Ras activation, we examined the brains of *PlpCre;Nf1fl*/*fl* mice exposed to tamoxifen at 2 months of age, and *CNP-HRas* mice (n = 5/genotype). Optic nerves were enlarged in *PlpCre;Nf1fl*/*fl*, and *CNP-HRas* mice at 12 months compared with WT animals (Figures 1A and 1B; quantification in Figure S3; p < 0.001), and the corpus callosum was enlarged in the *PLPCre* model (Figures S3C and S3D; p = 0.8 × 10^−7^).

To explain the increases in optic-nerve size, we performed electron microscopy. In normal nerves, myelin sheath thickness is proportional to the diameter of a wrapped axon. Pathological changes in myelin thickness can be quantified via a g-ratio analysis. A g-ratio analysis shows the extent of myelin disruption and the prevalence of the myelin abnormalities in >5,000 axons per genotype, and unbiased counting of myelinated axons in optic-nerve sections revealed significantly shifted gratios after loss of *Nf1* or hyperactivation of HRas (Figure 1C). The g-ratios in mutant optic nerves shifted both to the right and down, indicating increased axonal diameter and myelin thickness in *Nf1*-deficient and *HRasG12V* mutant mice. In mutant animals, 35% of myelinated axons had g-ratios < 0.5; in contrast, 0.01% of WT fibers had g-ratios < 0.5. Analysis of electron micrographs showed that myelin in mutants was expanded due to splitting/decompaction of myelin lamellae rather than an increase in the number of lamellae. In cross-section, compact myelin is composed of major dense lines (compressed cytoplasm) and intraperiod lines composed of adjacent plasma membranes (see model in Figure 1D). After *Nf1* loss or HRas-GTP expression in oligodendrocytes, the intraperiod lines were separated by extracellular space (Figure 1E).

Myelin decompaction can occur due to alterations in myelin, TJ, and/or GJ proteins. The brain myelin proteins 2′,3′-cyclic nucleotide 3′-phosphodiesterase (CNP) and PLP showed no significant changes in concentration (Figure 1F). The myelin basic protein (MBP) protein 24 kDa band decreased with a corresponding increase in 14 and 18 kDa bands, possibly reflecting changes in isoform expression. TJs span compact myelin layers (see schematic in Figure 1D). Claudin-11 levels in optic-nerve lysates were reduced after *Nf1* loss or *HRasG12V* expression (Figure 1G).

Because claudin-11 loss alone is insufficient to explain the observed myelin decompaction, we analyzed the GJ protein connexin 32 (Cx32), which is normally found at myelin interperiod lines (Ahn et al., 2008). Cx32 messenger RNA (mRNA) was unchanged (not shown). Cx32 protein was increased in optic-nerve lysates after *Nf1* loss or hyperactivation of HRas (Figure 1H). To define Cx32 distribution, we fractioned brain membranes by density centrifugation. In WT brains, Cx32 was enriched in myelin, whereas after *Nf1* loss or *HRasG12V* expression, Cx32 was concentrated in plasma membrane/endosomal fractions (Figure 1I). Band shifts characteristic of phosphorylated and dimerized Cx32 proteins were also present in mutant cytoplasmic fractions.

Correlates of increased axon diameter can include alterations in mitochondria in affected axons. Confirming our g-ratio analysis, electron microscopy displayed enlarged axons that may contribute to enlarged white matter tracts (Figure S4A). Because Ras has been shown to directly regulate mitochondria, it is interesting to note that in some axons in *PLP; Nf1floxed* (not shown) or *CNP-HRas* animals, mitochondria were more numerous, present near noncompact myelin, or showed abnormal cristae (Figures S4B and S4C). Neuronal cell death, as indicated by cleaved caspase3+ in cell bodies, was present in the retinal ganglion cell layer of mutants (Figure S4D).

### GJs Are Lost in Enlarged Astrocyte Endfeet after *Nf1* Loss or HRas Activation

**Figure 2:**
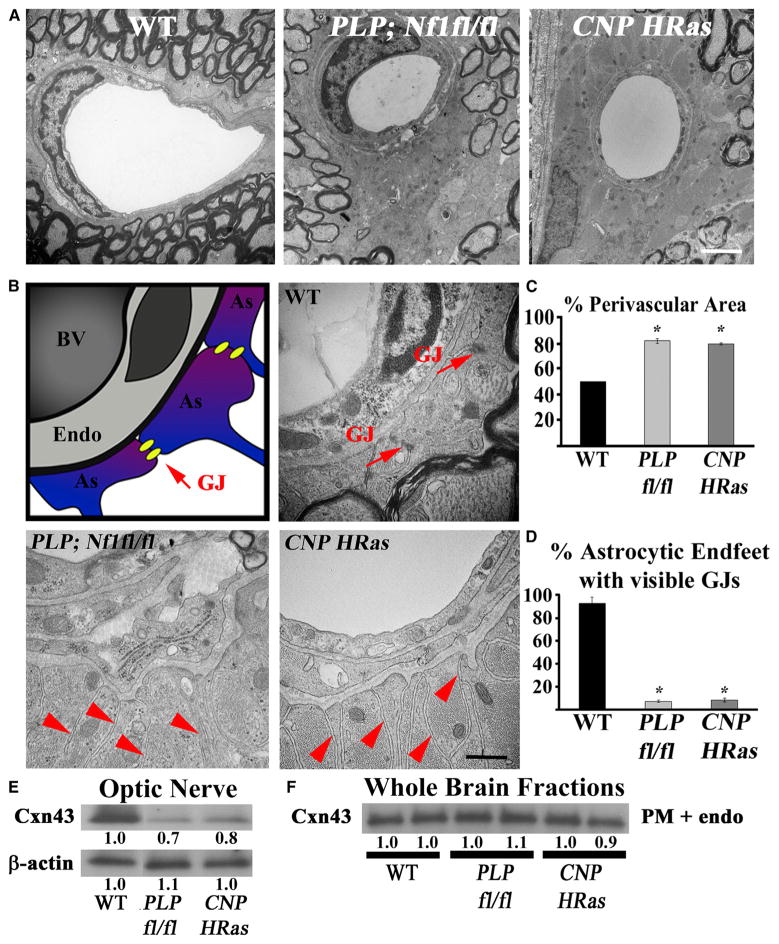
GJs Are Lost in Enlarged Optic-Nerve Astrocyte Endfeet after *Nf1* Loss or HRas Activation (A) Electron micrograph cross-sections of capillaries and perivascular space in optic nerve 1 mm from the chiasm (10,000×; scale bar, 2 μm). (B) Left: Diagram of capillary. Yellow: GJs. As, astrocyte endfoot; BV, blood vessel; Endo, endothelial cell. (B) Electron micrographs of capillaries 1 mm from the chiasm (50,000×; scale bar, 250 nm). Red arrows: GJs on astrocyte endfeet; red arrowheads: astrocyte endfeet lacking GJs. (C) Quantified perivascular area (% total area divided by vascular area). (D) Percentage of astrocyte endfeet with visible GJs. (E and F) Western blots using total optic-nerve lysate (E) or whole-brain endosomal fractions (F) showing Cx43. n = 3–5 animals/genotype. *p < 0.01.

Increased myelin sheath diameters partially accounted for optic-nerve enlargement. Electron microscopy also revealed aberrantly wide perivascular space surrounding the capillaries in mutant optic nerves (Figure 2A; quantification in Figure 2C). The expanded perivascular space contained enlarged astrocyte endfeet surrounding endothelial cells (Figure 2B). These swollen endfeet contain increased numbers of mitochondria (not shown), indicating a possible shift in metabolic load at these sites. In WT brains, adjacent astrocyte endfoot membranes contain GJs composed of Cx43 (Simard et al., 2003). GJs appear as characteristic electron-dense plaques at opposed astrocyte membranes (Figure 2B, red arrows: GJ). In contrast to the WT brain, electron-dense plaques were rarely observed in astrocyte end-feet surrounding optic-nerve capillaries after *Nf1* loss or HRas activation (Figure 2B, arrowheads; quantification in Figure 2D).

Reductions in visible GJs at astrocyte endfeet could result from changes in Cx43, which is astrocyte specific in the brain. Quantitative RT-PCR did not show changes in Cx43 mRNA expression (not shown). Cx43 protein was decreased in *PLP;Nf1fl*/*fl* or *CNP-HRas* animals’ optic nerves (Figure 2E), but was not detectably changed in whole-brain lysates (Figure 2F). Decreased Cx43 was detectable in oligodendrocyte-rich myelin tracts, where *Nf1*/*Ras* mutant cells are enriched.

### *Nf1* Loss or HRas Activation in Oligodendrocytes Results in Changes in Endothelial Claudins and BBB Permeability

**Figure 3:**
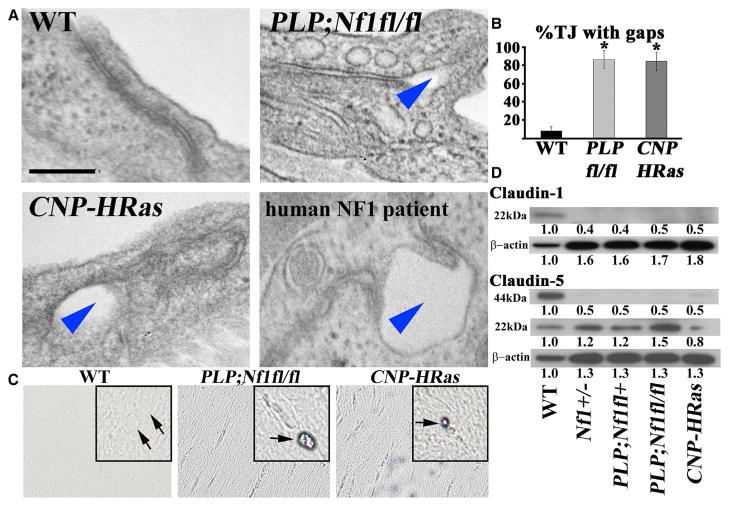
*Nf1* Loss or HRas Activation in the Optic Nerve Causes Changes in Claudins and BBB Permeability (A) Electron micrographs of optic-nerve cross-sections of capillaries 1 mm from the chiasm (50,000×; scale bar, 200nm) show electron-dense TJs between endothelial cells. Blue arrowheads: areas of TJ disruption. (B) Quantification of the total TJs with gaps, with 200–300 TJs counted per genotype. (C) Evans blue stain of longitudinal sections of *PLPCre; Nf1fl*/*fl* and *CNP-HRas* optic nerve. Insets: cross-sections; arrows: blood vessel. (D) Western blots from total optic-nerve lysates showing claudin-1 and claudin-5. All experiments included three to five animals per genotype. *p < 0.001. See also Figure S5.

Astrocytes induce endothelial cells to form TJs, which are critical for the formation of the BBB (Wolburg and Lippoldt, 2002). TJs appear as continuous electron-dense plaques between endothelial cells in transmission electron micrographs (Figure 3A, top left). After *Nf1* loss or *HRasG12V* expression, TJs were present between endothelial membranes but contained gaps. We found similar gaps in electron micrographs of optic-nerve blood vessels in a human NF1 autopsy sample (Figure 3A, blue arrowheads; quantification in Figure 3B). The use of human tissue was approved by the institutional review board of the Cincinnati Children’s Hospital.

To test whether loss of astrocyte endfoot GJs and endothelial TJ malformations result in functional alterations in the BBB, we injected Evans blue dye into WT, *PlpCre;Nf1fl*/*fl*, and *CNP-HRas* mice. As expected, 24 hr later the WT animals displayed no Evans blue leak. Strikingly, both *PlpCre;Nf1fl*/*fl* and *CNP-HRas* optic nerves showed residual dye in the perivascular space (Figure 3C). No detectable Evans blue dye was found in the parenchyma, indicating that leakage, but not extensive BBB disruption, occurred in the capillaries. Western blot analysis showed downregulation of the BBB TJ protein claudin-1 and loss of claudin-5 dimer after *Nf1* loss or *HRasG12V* expression (Figure 3D), confirming endothelial TJ impairment. Vasculature abnormalities must be non-cell autonomous, as neither the *PlpCre* driver nor *CNP-HRas* was expressed in astrocytes or endothelial cells.

### Optic-Nerve Enlargements Are Not Gliomas

NF1 patients are predisposed to grade 1 pilocytic astrocytomas, which are frequent along the visual pathway and are characterized by optic-nerve enlargement, astrocyte proliferation, angiogenesis, and deposition of Rosenthal fibers and eosinophilic granular bodies (Perilongo et al., 2012). None of these features were present after *Nf1* loss or *HRasG12V* expression in oligodendrocytes. GFAP immunohistochemistry of optic-nerve cross-sections revealed no reactive or neoplastic astrocyte cell proliferation up to 12 months after tamoxifen injection (Figure S5A). Although there was a significant increase in the number of blood vessels per optic-nerve cross-section in the *PLP;Nf1fl*/*fl* and *CNP-HRas* mice (not shown), it was not significant after normalization to the nerve area (Figure S5B). The cellularity of optic-nerve cross-sections in H&E-stained paraffin sections did not change, and there was no increase in cell proliferation as assessed by BrdU or Ki67/EGFP+ immunohistochemistry (not shown). H&E analysis of optic-nerve sections (cross and longitudinal) revealed one case of focal, infiltrating optic glioma without the presence of inflammatory cells or increased reactive gliosis (not shown); however, this was very rare (n = 1/60).

Despite the absence of proliferating EGFP+ cells at 12 months after tamoxifen injection, EGFP+ cells remained numerous in *PlpCre;Nf1fl*/*fl;EGFP+* animals, but diminished over time in *PLPCre;Nf1WT;EGFP+* optic nerve (Figure S5C). EGFP+ cells were present along the ventricular walls, where many migratory NG2+ progenitor cells are normally found. Immunohistochemistry revealed NG2+/EGFP+ double-positive cells in the subventricular zone of *PlpCre* animals (Figure S5D). This may explain the persistence of EGFP+ cells in the mutant optic nerves long after tamoxifen exposure, as NG2+, *Nf1+*/−, and *Nf1*−/− progenitors proliferate more than their WT counterparts and NG2+ progenitors migrate from the third ventricle to the optic nerves during development (Bennett et al., 2003; Hegedus et al., 2007).

### Myelin Decompaction and TJ and GJ Cellular Changes Resulting from Oligodendrocyte-Specific Ras Activation Are NO Dependent

**Figure 4:**
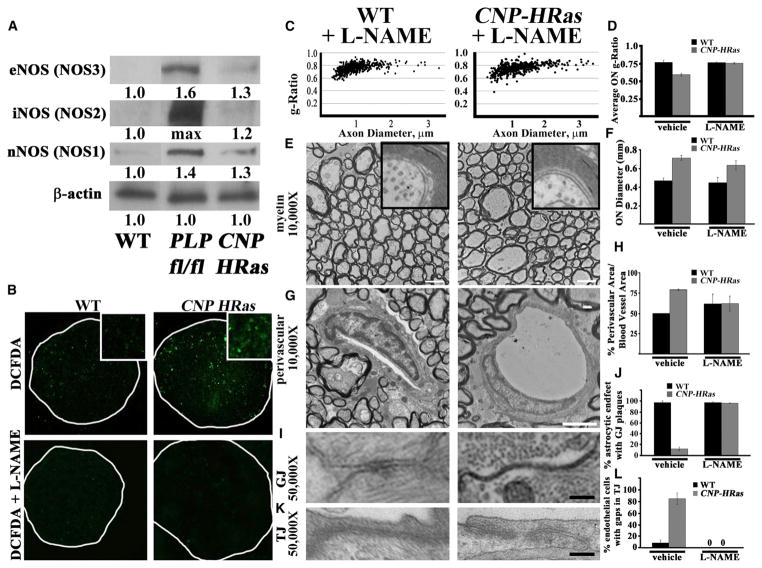
Increased ROS after *Nf1* Loss or HRas Activation NOS sufficiency for phenotype presentation. (A) Western blot analysis of NOS (NOS1–NOS3) in optic-nerve protein lysates. Blot intensity was measured with ImageJ software and caps at upper intensity levels; “max” indicates intensity beyond the upper intensity limitations. (B) DCF-DA stain in unfixed optic-nerve cross-sections (5×; inset: 40×) from WT and *CNP-HRas* mice with and without the NOS inhibitor L-NAME. White line shows optic-nerve boundaries. (C) g-Ratio scatterplots show measurements of >1,000 axons/animal. (D) Quantification. (E–K) Electron micrographs of optic-nerve cross-sections 1 mm rostral to the chiasm (E and G: 10,000× and scale bar, 2 μm; I and K: 50,000× and scale bar, 100 nm). (F) Optic area quantified in semithin cross-sections 1 mm rostral to the chiasm. (H, J, and L) Quantification of perivascular area (H), percentage of astrocyte endfeet with visible GJs (J,~300 GJs/animal), and percentage of endothelial cells with TJ gaps (L, ~50–60 TJs/animal) measured using electron microscopy; n = 3–5 animals/genotype. See also Figures S6 and S7.

The identification of non-cell-autonomous changes in astrocytes and endothelial cells after loss of *Nf1* or *HRasG12V* expression in oligodendrocytes prompted us to consider whether a secreted Ras-dependent effector molecule might be produced by mutant oligodendrocytes. We focused on ROS because increased ROS can affect the BBB (Komatsu et al., 1999), and ROS was implicated downstream of Ras (Ferro et al., 2012) and Nf1 (Tong et al., 2007). ROS include hydrogen peroxide (H_2_O_2_), superoxide O_2_−, and NO. NO is an important signaling molecule that is synthesized inside cells from L-arginine, oxygen, and nicotinamide adenine dinucleotide phosphate (NADPH) by mammalian NOS (NOS1, NOS2, and NOS3). Inside target cells, NO activates guanylate cyclase, oxidizes, ADP ribosylates, and nitrosylates proteins, DNA, and lipids. All three NOS proteins were upregulated in optic-nerve lysates after *Nf1* loss or *HRasG12V* expression (Figure 4A). ROS reaction products were also significantly increased in the CNP-HRas optic nerves (Figure 4B) and were diminished by preincubation with the NOS inhibitor NG-nitro-L-arginine methyl ester (L-NAME; Figure 4B, bottom), indicating the contribution of NO species.

To test whether the cellular phenotypes identified were caused by upregulation of NOS, CNP-HRas animals were administered L-NAME for 7 days and then the optic nerves were processed for electron microscopy. L-NAME rescued abnormal g-ratios (Figures 4C and 4D), perivascular area (Figures 4G and 4H), astrocyte GJs (Figures 4I and 4J), and endothelial TJs (Figures 4K and 4L). However, L-NAME administration for 7 days failed to rescue the increases in axon diameter (Figures 4E and 4F). Similar results were obtained in *PLP;Nf1fl*/*fl* animals (not shown).

### *Nf1* Haploinsufficiency in Oligodendrocytes Causes Myelin and Vascular Phenotypes

**Figure 5:**
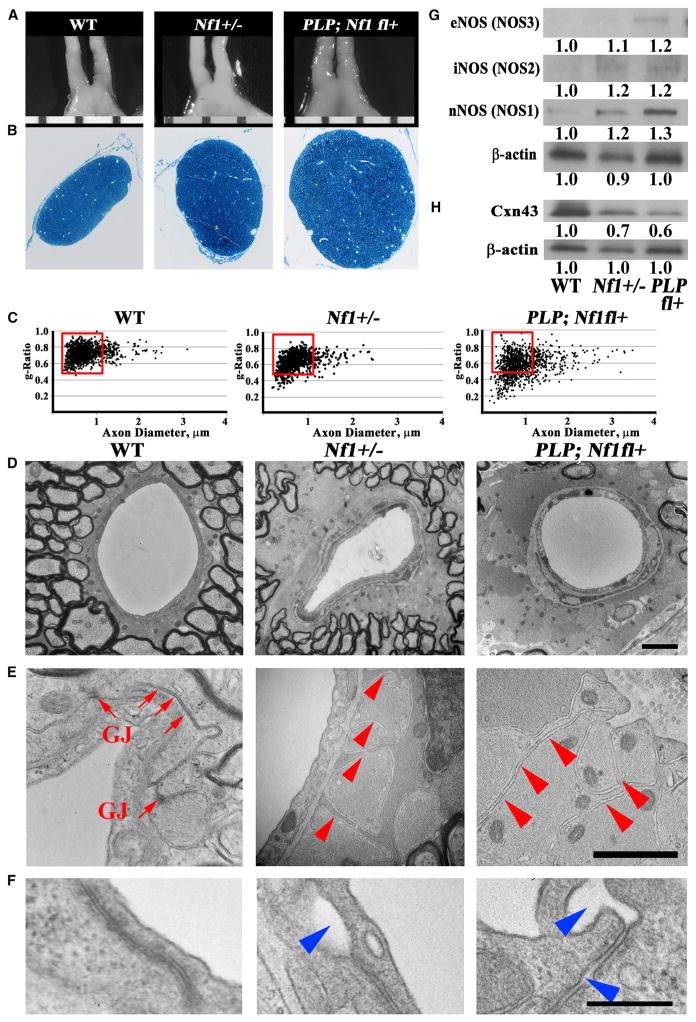
Heterozygous *Nf1* Loss in Oligodendrocytes Causes Myelin and Vascular Phenotypes (A) Gross micrographs of optic nerves at the level of the chiasm in 12-month-old animals. Ruler shows 1 mm markings. (B–F) Optic nerve 1 mm rostral to the chiasm. (B) Toluidine-blue-stained semithin cross-sections. (C) Scatterplots of g-ratios (~5,000 axons/graph) from three to five electron micrographs (10,000×) of three to five animals/genotype. Red box: 99% of WT g-ratios superimposed upon graphs of other genotypes. ANOVA, Tukey post hoc, p = 2.2 × 10^−16^. (D) Electron micrographs of capillaries and perivascular space (10,000×; scale bar, 2 μm). (E) Electron micrographs of optic-nerve cross-sections of capillaries (50,000×; scale bar, 200 nm). Red arrows: GJs on astrocyte endfeet; red arrowheads: astrocyte endfeet without GJs. (F) Electron micrographs of TJs between endothelial cells (50,000×, scale bar, 200 nm). Blue arrowheads: areas of TJ disruption. (G) Western blot analysis of NOS (NOS1–NOS3) in total optic-nerve lysates. (H) Western blots in total optic-nerve lysate showing Cx43.

Most NF1 patients are heterozygous for the *NF1* mutation; therefore, we examined whether heterozygous loss mimicked the myelin and/or vascular phenotypes identified in *PLPCre;Nf1fl*/*fl* and *CNP-HRas* models. Optic nerves were significantly enlarged in *PLPCre;Nf1fl*/*+* animals (Figures 5A and 5B; p < 0.0001), but not in *Nf1+*/− animals (p = 0.41). Both *Nf1+*/− and *PLPCre;Nf1fl*/*+* mice showed myelin g-ratio changes (Figure 5C), myelin decompaction with concurrent axonal enlargement (see Figure S7), Cx32 protein mislocalization (not shown), increased perivascular space with enlarged astrocyte endfeet (Figures 5D and 5E), and endothelial TJ disruptions (Figure 5F). On a molecular level, the heterozygotes showed increased NOS (NOS1–NOS3) protein expression in optic-nerve lysates (Figure 5G) and downregulation of optic-nerve Cx43 (Figure 5H).

### Myelin Decompaction and Perivascular Changes Are Also Found in the Corpus Callosum after Nf1 Haploinsufficiency or HRas Activation

The corpus callosum is enlarged in many NF1 patients. We examined electron micrographs from *Nf1+*/−, *PlpCre;Nf1fl*/*+*, *PlpCre;Nf1fl*/*fl*, and*CNP-HRas* animals, and found myelin decompaction at intraperiodlines, expanded perivascular space with loss of GJs at astrocyte end feet (blue arrows), and changes in endothelial TJ (not shown; Figure S6). The data were confirmed by g-ratio analysis (not shown). Thus, *Nf1* loss or *HRasG12V* in oligodendrocytes also affects hemispheric white matter. Corpus callosum size was measured from paraffin coronal sections at the level of rostral anterior commissure from three to seven animals per genotype. The corpus callosum of *PlpCre;Nf1fl*/*+* and *PlpCre;Nf1fl*/*fl* animals was significantly increased when compared with WT controls (p = 0.6 × 10^−5^, p = 0.7 × 10^−6^, respectively; Figures S3C and S3D). Changes in the size of the corpus callosum in *Nf1+*/− and *CNP-HRas* mice were not significant.

### Generation of Increased ROS Is Intrinsic to Oligodendrocytes

**Figure 6:**
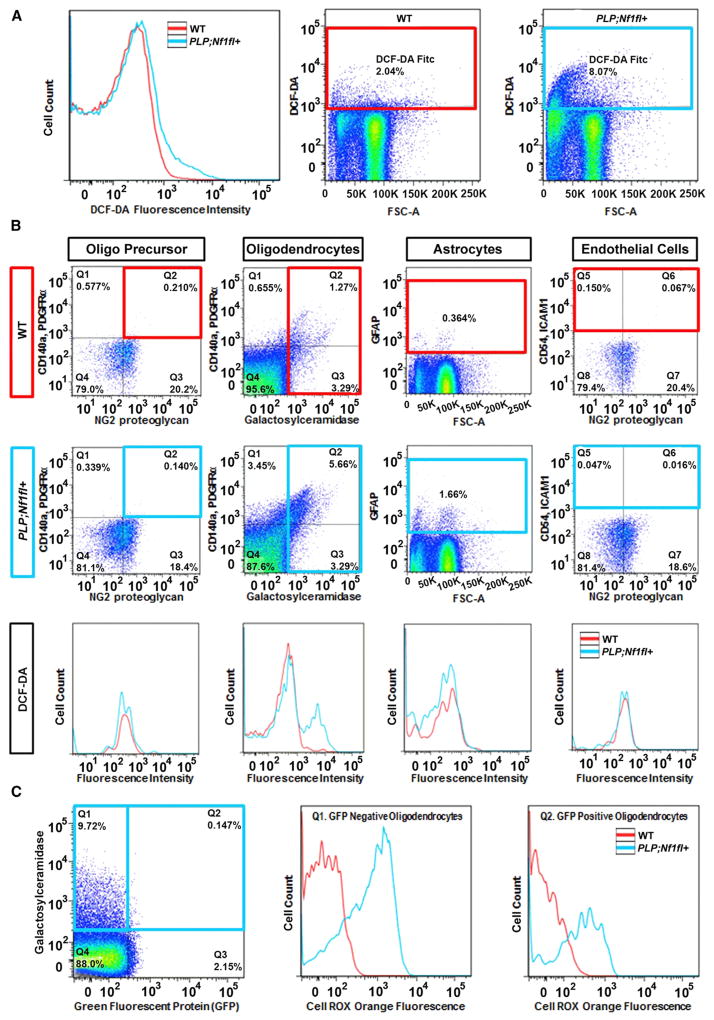
Increased Reactive Oxygen Is Detected Only in Oligodendrocytes (A) DCF-DA fluorescence intensity measured by flow cytometry in dissociated forebrain + optic nerves of WT and *PLP;Nf1fl+* mice 6 months after tamoxifen injection. Gates for DCF-DA with cell percentages indicated. (B) Gates were used to discriminate cell types. Top row: WT animals are shown with red gates. Middle row: *PLP;Nf1fl+* animals are shown with blue gates. Bottom row: DCF-DA fluorescence intensities per cell type with WT (red) and *PLP;Nf1fl+* (blue) lines. (C) Cell Rox Orange fluorescence in GFP− and GFP+ cells.

To determine which cell population(s) had increased ROS, we dissociated cells from the forebrain and optic nerve of *PLP;Nf1fl+;eGFP* mice (6 months after tamoxifen injection). We monitored the fluorescent ROS reporter 2′,7′-dichlorodihydrofluorescein diacetate (DCF-DA) by flow cytometry. There was no significant shift in DCF-DA fluorescence intensity (p = 0.74) or total live DCF-DA+ CNS resident cells (p = 0.51), although an increase was observed in some individual mutant mice (Figure 6A). We used gates to discriminate cell types (Figure 6B, top two rows). Importantly, a significant increase (p = 0.05) in DCF-DA fluorescence intensity was present in sorted GalC+ oligodendrocytes after *Nf1* loss (Figure 6B, bottom row). In contrast, platelet-derived growth factor receptor α (PDGFRα)+ progenitors, GFAP+ astrocytes, intercellular adhesion molecule 1 (ICAM1)+ endothelial cells, and CD45+ blood cells/microglia in the mutant brains showed similar ROS compared with WT cells. Because EGFP expression is Cre driven, most sorted EGFP+ cells from *PlpCre;Nf1fl*/*+* mice are *Nf1* mutant, and EGFP− cells are WT. Strikingly, there was a significant increase in Cell ROX Orange ROS reporter fluorescence intensity in both EGFP+ recombined (p = 0.01) and EGFP− (p = 0.001) GalC+ oligodendrocytes after *Nf1* loss (Figure 6C). The GFP− population was larger (p = 0.005) than that of GFP+ recombined cells, likely reflecting the larger number of total gated cells. Overall, the data indicate that *Nf1* loss results in ROS accumulation in mutant oligodendrocytes. ROS also accumulates in other oligodendrocytes, possibly those coupled directly by GJs.

### The Antioxidant N-Acetyl Cysteine Rescues Myelin and Vascular Phenotypes in *PLP;Nf1floxed* and *CNP-HRas* Mice

**Figure 7:**
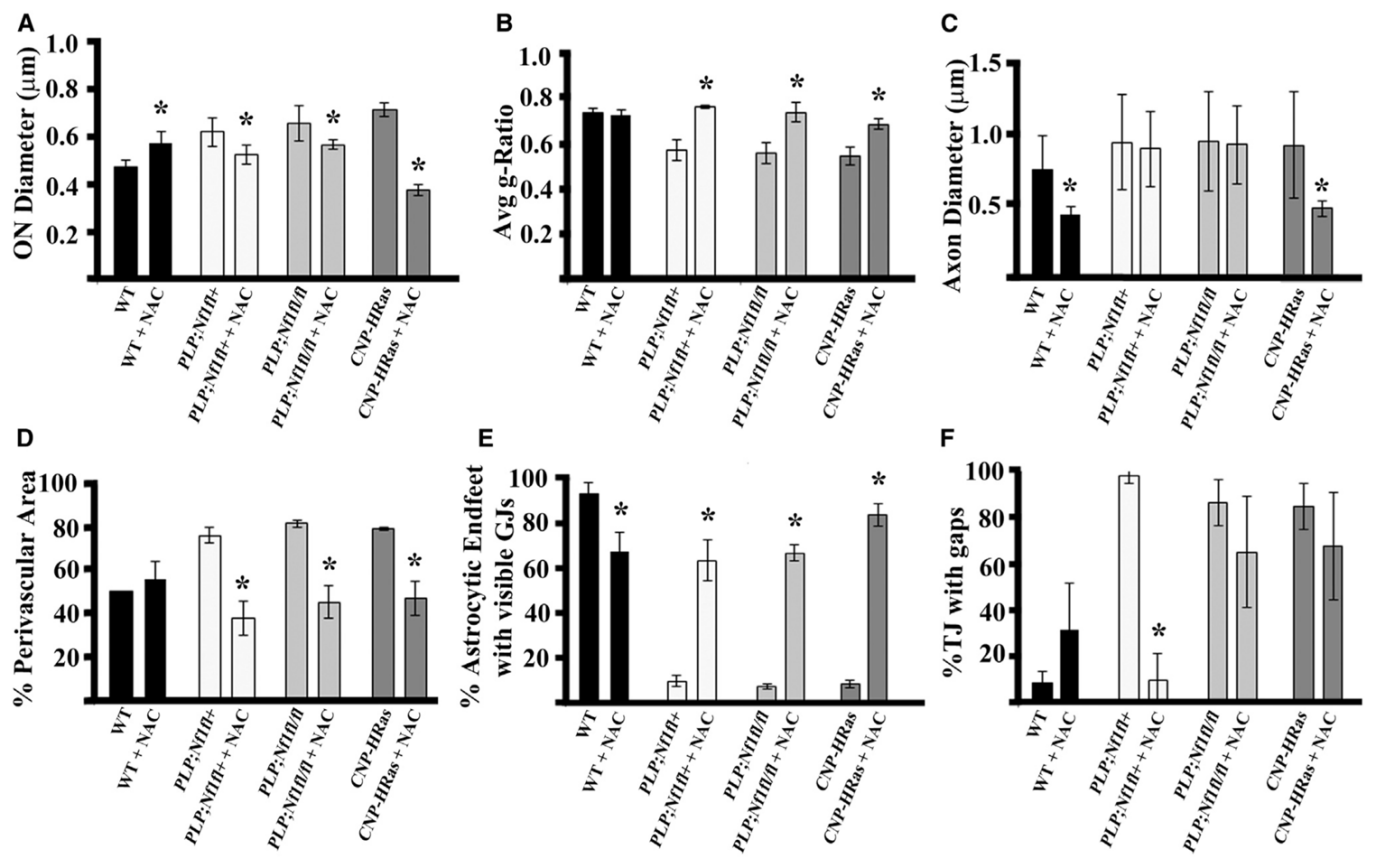
The Antioxidant NAC Rescues Myelin and Vascular Phenotypes in *PLP;Nf1floxed* and *CNP-HRas* Mice Quantification of phenotypes in WT, *PLP;Nf1fl+, PLP;Nf1fl*/*fl*, and *CNP-HRas* animals after 6 weeks of vehicle or NAC treatment, all in optic nerve 1 mm rostral to the chiasm. (A) Optic-nerve diameter quantified from semithin cross-sections. (B and C) Quantification of g-ratio (B) and axon diameter (C) of >1,000 axons/genotype measured by electron microscopy. (D) Quantification of % perivascular area normalized to blood vessel area. (E) Percentage of astrocyte endfeet with visible GJs (~300 GJs/animal). (F) Percentage of endothelial TJs with gaps (~50–60 TJs/animal); n = 3–5 animals/genotype.

Available inhibitors of NOS are broad spectrum and/or affect subsets of synthases. We tested a broad-spectrum antioxidant that is not specific for NOS, but can be used in vivo for prolonged periods. We administered the antioxidant N-acetyl cysteine (NAC; 0.001% in drinking water) to *PLP;Nf1fl*/*+, PLP;Nf1fl*/*fl*, and *CNP-HRas* animals. In the PLP models, NAC was administered 6–8 months after tamoxifen injection. In *CNP-HRas* mice, NAC was administered beginning at 10 weeks of age. In each case, phenotypes were rescued by 6 weeks of NAC exposure— completely in *CNP-HRas* mice and to a lesser extent in *Nf1* mutants. Optic-nerve diameter was rescued (Figure 7A) and g-ratios normalized in all strains (Figure 7B). Perivascular space was rescued (Figure 7D), as were GJs in astrocyte endfeet (Figure 7E). Myelin compaction was also rescued in all strains (Figure S7). Changes in axon diameter (Figure 7C) and TJ gaps (Figure 7F) varied among the animals and were not significant when compiled for *PLP;Nf1fl*/*+* or *PLP;Nf1fl*/*fl* mice; however, Evans blue staining showed no BBB leak in the optic nerve in any mutant model after NAC administration (not shown).

### *CNP-HRas* Animals Have Hyperactive Locomotion and Hypersensitivity to Startle

**Figure 8:**
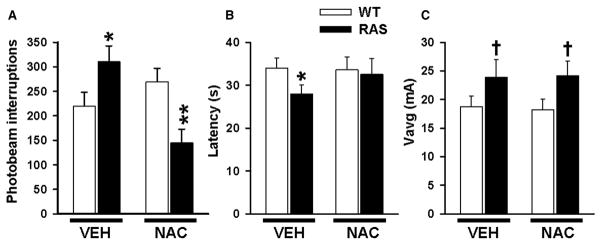
*CNP-HRas* Animals Have Hyperactive Locomotion and Hypersensitivity to Startle Behaviors Quantification of behavior in *CNP-HRas* mice after 6 weeks of vehicle or NAC treatment. (A) Peripheral beam interruptions on locomotor behavioral test. *Vehicle p < 0.05; **NAC p < 0.01. (B) Mouse swim latency to get to the cued platform during day 1 of the Morris water maze task. *Vehicle p < 0.05; NAC p > 0.08. (C) Average startle Vavg. † Vehicle p = 0.07; NAC p < 0.07; n = 15/genotype/treatment.

Changes in myelin have recently been correlated with changes in behavior, and NF1 and Costello syndrome patients are known to display frequent behavioral deficits; therefore, we tested whether behavior was altered in mice with elevated Ras-GTP in oligodendrocytes. Because the *PLP;Nf1flox;eGFP* mouse lines were on a mixed strain background, we focused behavioral experiments on the *CNP-HRas* animals on a pure C57Bl/6 background. *CNP-HRas* animals were hyperactive in a locomotor task (Figure 8A, p = 0.045) and swam faster in the cued Morris water maze (Figure 8B, p = 0.01) as compared with littermate controls. They also showed a trend toward a hyperreactive startle response (Figure 8C, p < 0.07). No significant differences were noted in parallel bars, tremor, or in the learning and memory phases of the Morris water maze (not shown). NAC exposure increased locomotor activity in the WT mice, correlating with myelin decompaction, GJ and TJ changes, and Evans blue BBB leak (not shown), indicating that high antioxidant exposure in normal animals without high ROS levels can be detrimental. In contrast, 6 weeks of NAC treatment decreased locomotor activity in the *CNP-HRas* mice by 30.8%, bringing them below control levels (p < 0.01). NAC normalized swim speed in the cued Morris maze, but did not modify the startle response in these mice. Thus, behavioral changes induced by Ras upregulation in oligodendrocytes are partially reversible by antioxidant therapy.

## DISCUSSION

We generated a *CNP-HRasG12V* mouse model to examine the role of HRas activation in oligodendrocytes. A striking gross abnormality in Ras mutant brain was enlargement of optic nerves. This finding extended to the *PLPCreERT;Nf1floxed* mouse models, consistent with the idea that the cellular effects of *Nf1* loss of function in adult myelin are mediated through Ras activation. This enlargement of white matter is consistent with previously reported white matter enlargement and macrocephaly in NF1 patients, which lacked a cellular or molecular explanation (Pride et al., 2010; Karlsgodt et al., 2012). We find that Ras pathway activation in oligodendrocytes upregulates NOS and elevates oligodendrocyte ROS, including NO. NO modulates TJ and GJ proteins in oligodendrocytes and in surrounding astrocytes and endothelial cells, affecting brain permeability and contributing to behavioral abnormalities. These data provide a potential cellular and molecular mechanism of Rasopathy brain abnormalities. It will be of interest to closely examine the brains of Rasopathy patients for white matter enlargement and the cellular features we have identified.

Using inducible *Plp-Cre*, we targeted loss of *Nf1* to mature adult oligodendrocytes. Similar phenotypes developed as compared with the *CNP-HRas* model in which oligodendrocytes were affected beginning during development. Given our ability to rescue phenotypes in both models during adulthood, the data argue that these brain defects are not developmental in origin. These data are consistent with findings that learning defects can be rescued in adult mice with *Nf1* mutations (Li et al., 2005). Wang et al. (2012) rescued enlarged corpus callosum size with MEK inhibitors administered from birth to postnatal day 18. Our data support the idea that critical defects can be rescued after they form, even into adulthood.

Some differences between the *Plp-Cre;Nf1fl*/*fl* and *CNP-HRas* models were observed. Although all cellular phenotypes were detected in both models, the corpus callosum was not significantly enlarged in the *CNP-HRas* mice. In Costello syndrome, the brain can atrophy. It remains possible that the size of the corpus callosum in our mouse model changed with the age of animals studied. Also, although NAC exposure rescued g-ratios, GJs, and perivascular abnormalities in both models, axon diameter was rescued in the *CNP-HRas* model but not in the *Plp-Cre;Nf1* model. These differences may be caused by the fact that H-Ras is constitutively GTP loaded in *CNP-HRas* oligodendrocytes, whereas Ras loading in *Nf1* mutants, including oligodendrocyte lineage cells, can have elevated basal Ras-GTP yet also maintain cytokine/growth factor responsiveness (Bennett et al., 2003). In addition, loss of Nf1 protein is predicted to activate all Ras proteins, whereas only H-Ras should be activated in *CNP-HRasG12V* mice.

Patients with NF1 are predisposed to develop grade 1 pilocytic optic-nerve gliomas. In humans, and especially in NF1 patients, some pilocytic tumors grow while others do not. At present, MRI optic-nerve enlargement alone is diagnostic for optic glioma in NF1 patients (Perilongo et al., 2012). Our results identify a potential mechanism that may underlie optic-nerve enlargement in some patients who do not progress to tumors, and suggest that optic-nerve enlargement may not be sufficient for glioma diagnosis. The use of an antioxidant might provide therapeutic options to these patients and at least partially reduce nerve size.

*Nf1* loss or HRas activation in oligodendrocytes did not cause optic glioma, and the mice did not display cytological atypia or increased astrocyte proliferation characteristic of pilocytic astrocytoma. NG2-Cre-mediated inactivation of *Nf1* also did not cause mouse optic glioma (Solga et al., 2013). Our *PLPCre; Nf1floxed* and *CNP-HRas* mouse models were similar to *GFAP-Cre; Nf1fl*/− mice, with optic-nerve enlargement and myelin disorganization (Kim et al., 2010). *GFAP-Cre;Nf1fl*/− mice have loss of *Nf1* in most or all of their brain cells. The enlarged optic nerve and disruption of myelin sheaths that are features of all three models are likely caused by defects in oligodendrocytes. Our identification of cell-autonomous *Nf1*/Ras contributions in oligodendrocytes represents a first step in defining how specific cell types contribute to optic-nerve enlargement in NF1 patients.

Changes in the brain structure of NF1 patients can be global, and possibly caused by mutation in a single *Nf1* allele (van Engelen et al., 2008; Karlsgodt et al., 2012). It is therefore relevant that myelin decompaction, swollen perivascular astrocyte endfeet, and TJ integrity phenotypes were identified in *PLPCre;Nf1fl*/*+* and *PLPCre;Nf1fl*/*fl* mice. *Nf1+*/− mice also showed these phenotypes, although they were less frequent than in *PLPCre;Nf1fl*/*+*, *PLPCre;Nf1fl*/*fl*, or *CNP-HRasG12V* mice. *Nf1+*/− optic nerves also did not show significant enlargement. We speculate that *Nf1+*/− cells outside the oligodendrocyte lineage limit these phenotypes, possibly by scavenging excess extracellular reactive oxygen.

Cell-autonomous myelin decompaction correlated with a reduction in the TJ protein claudin-11 and mislocalization of the GJ protein Cx32. We identified Cx32 band shifts characteristic of phosphorylated and dimerized Cx32 proteins, which target Cx32 into endosomes and decrease GJ assembly (Segretain and Falk, 2004). Because claudin-11-KO alone is insufficient for myelin decompaction, our data support a role for the combined loss of claudin-11 and Cx32 in myelin decompaction.

Rasopathy patients, including those with Costello, Noonan, LEOPARD, and cardiofaciocutaneous (CFC) syndromes and NF1, can have heart and/or bone abnormalities (Tidyman and Rauen, 2008). Connexins are vital for normal heart and bone function (Radosinska et al., 2011; Saura et al., 2010; Zhang et al., 2006), and can regulate ROS transfer (Taniguchi Ishikawa et al., 2012). NO regulates Cx43 through transcriptional modulation and phosphorylation (Radosinska et al., 2011; Zhang et al., 2006). For example, NOS inhibition alters Cx43 concentration and GJ function in cardiomyocytes (Dlugosova et al., 2009), and NOS (eNOS and iNOS)-KO mice show bone and heart abnormalities (Afzal et al., 2004; Saura et al., 2010). Ras regulation of NOS (with changes in both connexins and claudins) may be relevant in multiple organ systems in Rasopathy patients.

TJs are present in compact myelin and between brain endothelial cells, where they form the BBB. We focused on the claudin family of TJ proteins in part because of their expression in peripheral nerve tumors from NF1 patients (Pummi et al., 2006). Claudins determine the selectivity of paracellular transport, and the barrier properties of claudins are regulated by environmental cues. NO can modulate claudin expression and subcellular targeting, and assembly of claudin-containing TJs (Wolburg and Lippoldt 2002; reviewed in Angelow et al., 2008). The specific properties of claudins modulated by Ras and *Nf1* in oligodendrocytes and endothelial cells remain to be studied.

By using a new method to dissociate living brain cells and analyze them by fluorescence-activated cell sorting (FACS), we showed that only GalC+ oligodendrocytes had elevated levels of ROS in mutant brains. The oligodendrocyte ROS likely contains NO, because L-NAME rescued DCF-DA reactivity in tissue sections and myelin compaction in vivo. L-NAME is an L-arginine analog, a competitive inhibitor of NOS that is widely used as a NO inhibitor in cell and animal studies. As with any inhibitor, off-target effects may limit interpretation. For L-arginine analogs, the described off-target effects are inhibition of L-arginine transport, resulting in NOS inhibition due to substrate depletion, and interaction with cellular iron complexes, as in NOS enzymes, which also result in NO depletion (Robertson et al., 1993). Finally, regarding specificity, we note that hyperoxia rescued L-NAME-induced limb deficits in an animal model, limb defects that can be mimicked by eNOS knockout (Tiboni et al., 2003). In this study, we were able to block DCF-DA fluorescence (which reads out reactive oxygen, NO, and reactive nitrogen species) using L-NAME in tissue sections from mutant mice, supporting our hypothesis that NOS and NO are relevant to the phenotypes described. However, roles for other ROS cannot be excluded by these studies. How oligodendrocyte ROS affects astrocytes and endothelial cells (which lack detectable increases in ROS) also remains unclear. Yet, L-NAME reversed GJ and TJ phenotypes in astrocytes and endothelial cells in vivo, indicating that blocking NO is sufficient to rescue these phenotypes. Extracellular NO can alter GJ function in astrocytes in vitro in an L-NAME-dependent manner (Gandhi et al., 2010). ROS may diffuse from oligodendrocytes to affect other cell types, although it is likely to diffuse only short distances before being reduced by contact with proteins and lipids. Alternatively, NO may diffuse from oligodendrocytes through GJs to neighboring astrocytes, yet remain below our level of detection in an astrocyte population analysis.

Viosca et al. (2009) found that mice with germline *G12VH-Ras* are hyperactive and show hypersensitivity. We found that *CNP-HRas* mice also show locomotor hyperactivity that can be rescued with antioxidants. Elevation of oxidative stress is known to cause locomotor hyperactivity in mice (Chen et al., 2012; Dumont et al., 2011). Increased reactive oxygen and impaired oxidative balance occur in patients with attention deficit hyperactivity disorder (Selek et al., 2012), suggesting that ROS may play a role in human hyperactivity. NO may also play a role in epileptiform hyperactivity, as anticonvulsants diminish NO levels (Vega Rasgado et al., 2011). It is therefore relevant that up to 60% of NF1 and Rasopathy patients present with hyperactivity (Acosta et al., 2012; Tidyman and Rauen, 2008).

Six weeks of NAC treatment increased locomotor activity in WT mice. Antioxidant treatment in WT mice also opened the BBB to Evans blue dye and caused some myelin decompaction with GJ and TJ changes (not shown), indicating that high antioxidant treatment in animals without high ROS levels can be detrimental. These data support our finding that reactive oxygen balance is important for the cellular phenotypes identified, and suggest that antioxidant treatment could be used to temporarily open the BBB.

In summary, we have identified a pathway in which increased Ras-GTP in oligodendrocytes, through NOS/ROS, disrupts myelin compaction, TJ and GJs, and BBB permeability, with behavioral consequences. The relationship among these phenotypes and the potential for antioxidant therapies for Rasopathy patients will be important subjects for future studies.

## EXPERIMENTAL PROCEDURES

### Mice

*PlpCreERT; Nf1fl*/*+; eGFP* and *PlpCreERT; Nf1fl*/*fl; eGFP* mouse husbandry, tamoxifen injections, tissue processing, histology, and electron microscopy were performed as previously described (Mayes et al., 2011). WT and *Nf1*+/− were mice backcrossed for >30 generations onto a C57Bl6 background and genotyped by PCR (Brannan et al., 1994). Transgenic *HRASG12V* mice were created using a promoter from the CNP gene (Patel et al., 2012). Mice were backcrossed for more than eight generations onto a C57Bl/6 background and maintained on the male.

### Antibodies

Cryostat sections were processed for immunohistochemistry and imaged as previously described (Mayes et al., 2011) with markers for EGFP (GFP 1:2,000; Millipore), oligodendrocytes (Olig2 1:500, Millipore #AB9610; CC1 1:2,000, Calbiochem), progenitor cells (NG2 1:500, Millipore #AB5320), astrocytes (GFAP 1:500, Millipore #MAB360 or 1:1,000 DAKO), microglia (Iba1 1:2,000, Wako #019-19741), and neurons (NeuN 1:500, Chemicon #MAB377). Anti-Ki67 (1:250, Leica Novocastra #KI67P-CE) was used to determine cell proliferation. For HA staining, we used an anti-HA-epitope tag (1:200, Cell Signaling #2350S).

### g-Ratio Analysis

g-Ratios were determined by dividing the diameter of each axon by the diameter of the same axon together with its myelin sheath in 1,000–2,000 axons per animal (n = 3–5 per genotype) using ImageJ software.

### Western Blots

Optic nerves (n = 3–5 animals/genotype) were individually lysed and sonicated in 200 μl lysis buffer on ice and then clarified by centrifugation. Protein (30–50 μg) was separated by electrophoresis on SDS-polyacrylamide gradient gels (4%–20%; ISC BioExpress) and transferred to polyvinylidene difluoride membrane (BioRad). Membranes were probed with antibodies against myelin basic protein (SMI-99, 1:2,000, Calbiochem #NE1019), CNP (1:1,000, Cell Signaling Technologies 5664), PLP (1:2,000, Aves Labs), eNOS (1:1,000, Cell Signaling Technologies #9586S), iNOS (1:1,000, Cell Signaling Technologies #2977S), nNOS (1:1,000, Cell Signaling Technologies #4231S), connexin 32 (1:500, Invitrogen #138200), connexin 43 (1:100, Invitrogen #710700), claudin-1 (1:200, Invitrogen #71-7800), claudin-5 (1:1,000, Invitrogen #34-1600), and claudin-11 (1:1,000, Invitrogen #36-4500). They were then reprobed with anti-β-actin (1:5,000, Cell Signaling Technologies #4967) or MEK1/2 (1:1,000, Cell Signaling Technologies #4694S) as loading controls. Digitized blots were inverted and band intensities were quantified using ImageJ software.

### Ras Activation Assay

Adult brains were homogenized on ice and levels of activated Ras were determined using a Ras activation assay kit (Upstate Biotech) in which Ras-GTP was assayed using the GST-tagged Ras-binding domain of Raf1 (Raf-RBD) as an affinity reagent.

### Myelin Fractionation

The animals were euthanized and fresh brains were dissected immediately on ice, weighed, finely minced, and homogenized (1 g tissue/10 ml buffer: 0.32 M sucrose, 1 mM EDTA, 8.5 μM leupeptin, 1 μM phenylmethyl sulfonyl fluoride) with a dounce homogenizer (seven strokes each, with first a loose and then a tight pestle). Homogenates were spun in an Eppendorf 5702R centrifuge at 1,000 *g* for 15 min at 4°C. This total lysate was spun in a BeckmanCoulter Optima L-look ultracentrifuge at 10,000 *g* for 30 min at 4°C, and the supernatant from this spin was respun at 100,000 *g* for 1 hr at 4°C to generate cytosol. Total lysate was layered over steps of 0.32, 0.85 M, and 1.2 M sucrose, and spun in a BeckmanCoulter Optima L-look ultracentrifuge at 75,000 *g* for 30 min at 4°C. Myelin membranes were collected at the 0.32 M/0.85Msucrose interface with a Pasteur pipette. Plasma membrane was collected at the 0.85 M/1.2 M sucrose interface, and 30 μg of protein was loaded per lane for western blot analysis.

### ROS

Unfixed optic nerves were frozen in optimal cutting temperature (OCT) medium, and 12 μmcryostat sections cut and stored at −80°C. After air drying and rinsing, we incubated sections in 4 μM DCF-DA (5-(and-6)-chloromethyl-DCF-DA, acetyl ester, Invitrogen/Molecular Probes, #C6827) for 30 min at 37°C in PBS in a 5% CO_2_ incubator. Some sections were incubated with NOS inhibitor L-NAME (Sigma; 100 μMin PBS) for 60 min at room temperature before incubation with DCF-DA. Slides were rinsed with PBS and coverslipped. Green fluorescence was captured on a Zeiss Axiovert 200M microscope using a 40× Plan-Neofluar objective, Hamumamatsu Orca ER camera, and ImageJ software.

### Evans Blue Stain for BBB Function

Mice were given 4 ml/kg i.p. Evans blue (2% in PBS). After 24 hr, the mice were perfused with 100 ml ice-cold PBS and their brains were dissected. Tissues were fixed in 4% paraformaldehyde overnight and then placed into 20% sucrose for cryostat sectioning and imaging.

### Flow Cytometry

Brains were perfused with 30 ml PBS and then dissected, roughly chopped, and incubated in 1 ml Accutase (Millipore) for 30 min at 37°C. After addition of 10% fetal bovine serum (FBS) in Hank’s balanced salt solution (2 ml), the brains were manually triturated with a transfer pipette (~20×), transferred to cell strainers (100 μm; BD), and gently processed through the filter using 3 ml syringe plungers. Cell suspensions were centrifuged (3 min × 480 *g*), washed with 10 ml buffer (DPBS, 2% FBS, 2 mM EDTA), and recentrifuged. To purify cells from myelin debris, cells were resuspended in 40% Percoll (Amersham) and centrifuged at 650 *g* for 25 min at room temperature. The myelin top layer was aspirated and mononuclear cells were resuspended in buffer. Fc receptors were blocked by incubation with anti-mouse CD16/32 (1 μg/sample; eBioscience) on ice for 10 min. After washing (2×) in buffer and incubation in primary antibodies for 1 hr at 4°C, the cells were washed (3×) in buffer and resuspended in 200 μl buffer for analysis. Flow cytometry was performed on a FACS Cantos II flow cytometer using violet (405 nm), red (633 nm), and blue (488 nm) lasers. Antibodies for oligodendrocytes (GalC 1:200; Millipore), progenitor cells (PDGFRα 1:100 and NG2 1:400; Millipore), astrocytes (GFAP 1:200; BD Biosciences), endothelial cells (ICAM-1/CD54 1:400; Biolegend), and blood/microglia (CD45 1:200; BD Biosciences) were used. DCF-DA Fitc (1:200; Invitrogen) and Cell ROX Orange (1:1,000; Invitrogen) stained for ROS. Fixable dead cell stain (1:1,000; Invitrogen) was also used.

### Behavior

Behavioral assessments were completed on 3- to 4-month-old male *CNP-HRas* animals. Locomotor, startle, Morris water maze, and parallel bar tasks were assessed as previously described (Skelton et al., 2011; Vorhees and Williams, 2006; Kamens and Crabbe, 2007). Tremor was assessed in a San Diego Instruments SR-LAB apparatus with optional tremor detection capability for 20 min.

### NAC

NAC (1 g/l = 0.001%) was made fresh every other day in water and provided in the animals’ drinking water.

### Statistical Analyses

Counting was performed on 150–300 EGFP+ cells/area/animal in three to five animals/genotype. Single comparisons between littermates were done via Student’s t tests. For comparisons of groups, one-way ANOVAs followed by Tukey post hoc tests were performed using a significance cutoff of p < 0.05. Locomotor activity, Morris maze, and acoustic startle were initially analyzed by mixed linear ANOVA with factors of genotype and interval (for activity), day (for Morris maze), or trial (for acoustic startle). Follow-up comparisons for genotype differences averaged across interval, day, and trial were compared by t test for independent samples (two-tailed).

## Supplementary Material
