# Supplementary material for: Nf1 Loss and Ras Hyperactivation in Oligodendrocytes Induce NOS-Driven Defects in Myelin and Vasculature
Source: Cell Rep. Author manuscript; Available in PMC 2014 Apr 10. (PMC3982616; doi:10.1016/j.celrep.2013.08.011)
Supplement: Supplementary file 1 [file NIHMS567618-supplement-supplement_1.pdf]

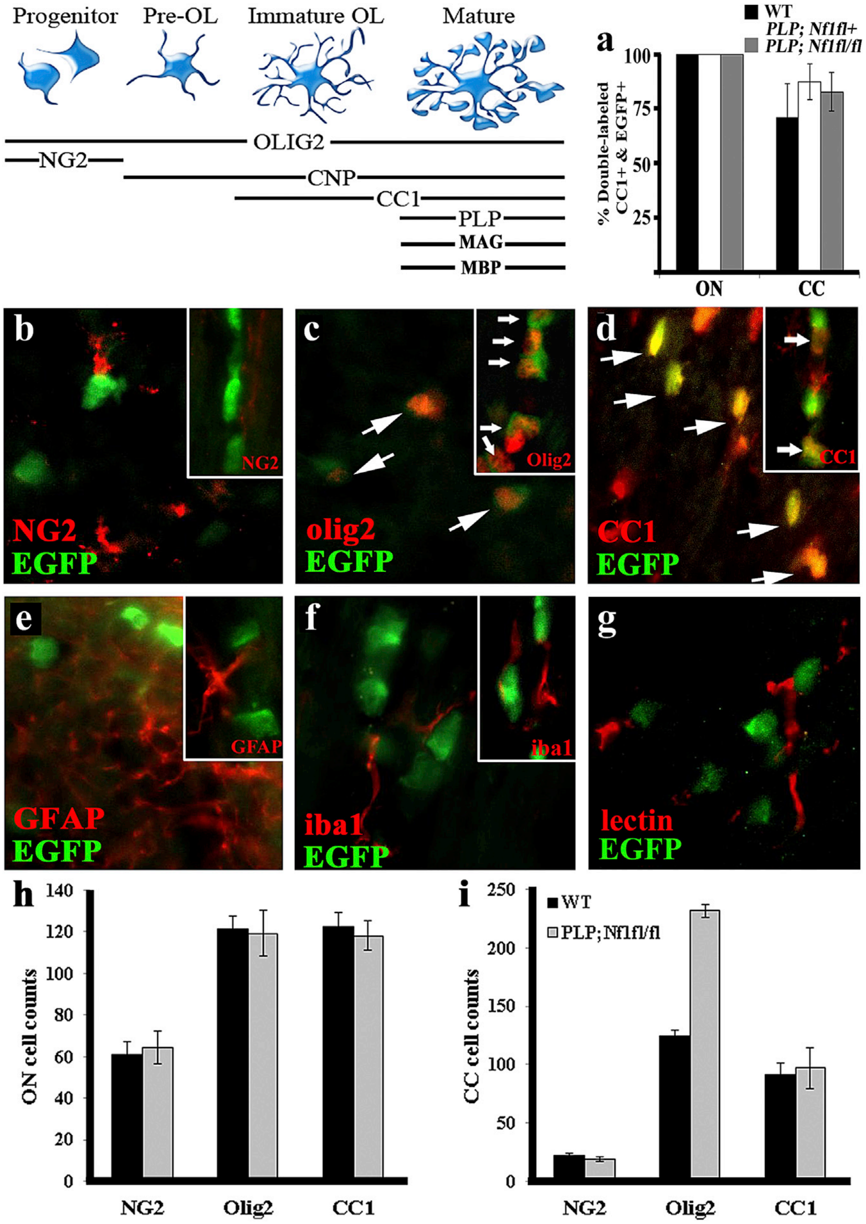

Figure S1. PLPCreERT Model System, Related to Figure 1

PlpCre targets mature oligodendrocytes in the central nervous system: identification using EGFP.

Top: Diagram of markers throughout oligodendrocyte development.

(A) Graph indicates percentages of recombined EGFP+/CC1+ double-labeled mature oligodendrocytes in optic nerve (ON) and corpus callosum (CC).

(B–G) Immunohistochemistry in optic nerve and corpus callosum (inserts) one day post adult Tamoxifen injections in *PlpCre;Nf1fl/fl* mice shows EGFP double labeled with NG2+ progenitor cells (B), olig2+ (C) and CC1+ (D) mature oligodendrocytes, GFAP+ astrocytes (E), Iba1+ microglia (F), and tomato-lectin+ endothelial cells (G). Arrows highlight double-labeled cells. n = 3-5.

(H and I) Quantification of oligodendrocyte lineage cells in the opic nerve (ON) and corpus callosum (CC) of wild type and PlpCre;Nf1fl/fl mice (n = 3-5 animals per genotype per marker).

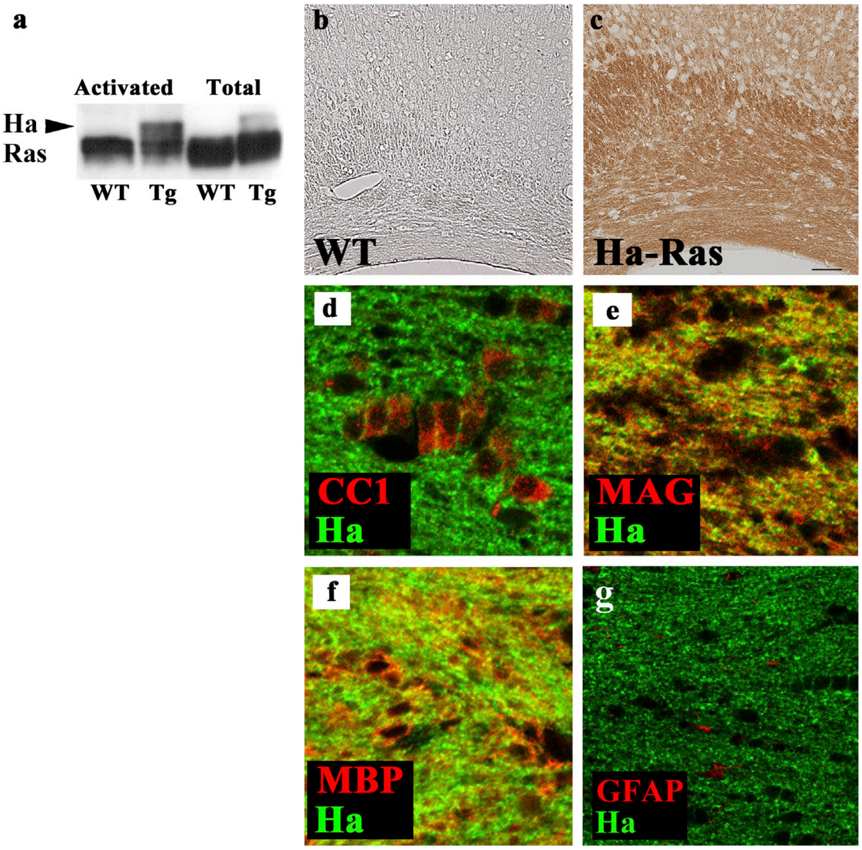

Figure S2. ***CNP-HRasG12V*** **Mouse Model, Related to Figure 1**
CNP targets mature oligodendrocytes in the central nervous system: identification using an HA tag.
(A) Western blot showing activated mutant HRas in the corpus callosum of *CNP-HRas* mice.
(B and C) Staining for the HA-tagged mutant HRas construct in (B) wild-type (WT) and (C) *CNP-HRas* corpus callosum.
(D–G) Immunohistochemistry in *CNP-HRas* mouse corpus callosum showing HA-tag staining double-labeled with CC1+ (D), MAG+ (E), and MBP+ (F) mature oligodendrocytes or GFAP+ astrocytes (G). n = 3-5 animals/genotype.

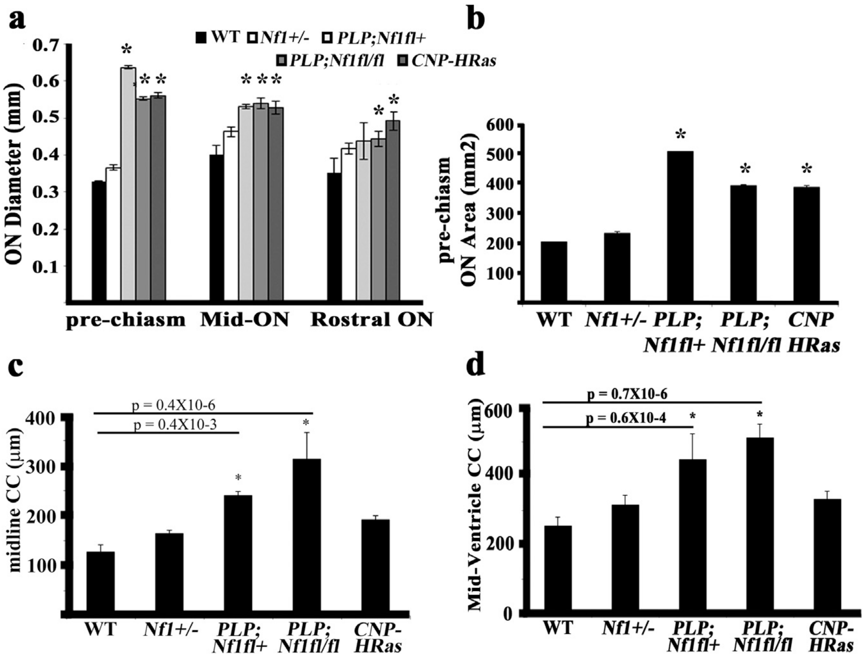

Figure S3. Measurements of Optic Nerve Enlargements, Related to Figure 1

(A) Average optic nerve diameter (n = 5/genotype) measured from gross dissected micrographs at the pre-chiasm (1mm rostral to the chiasm), Mid-ON (2 mm rostral to the chiasm), and Rostral ON (4mm rostral to the chiasm). *ANOVA, Tukey post hoc, $p < 0.0003$.

(B) Average area of optic nerve semithin crossections 1mm rostral to the chiasm (n = 5/genotype). *ANOVA, Tukey post hoc, $p < 1X10^{-6}$.

(C and D) Average corpus callosum size (n = 7/genotype). Measurements were taken at (C) the midline *ANOVA, Tukey post hoc, $p = 8.3x10^{-7}$ or (D) the midpoint of lateral ventricle *ANOVA, Tukey post hoc, $p = 8.1x10^{-7}$ at the level of the rostral part of the anterior commissure.

Cell PRESS
Open ACCESS

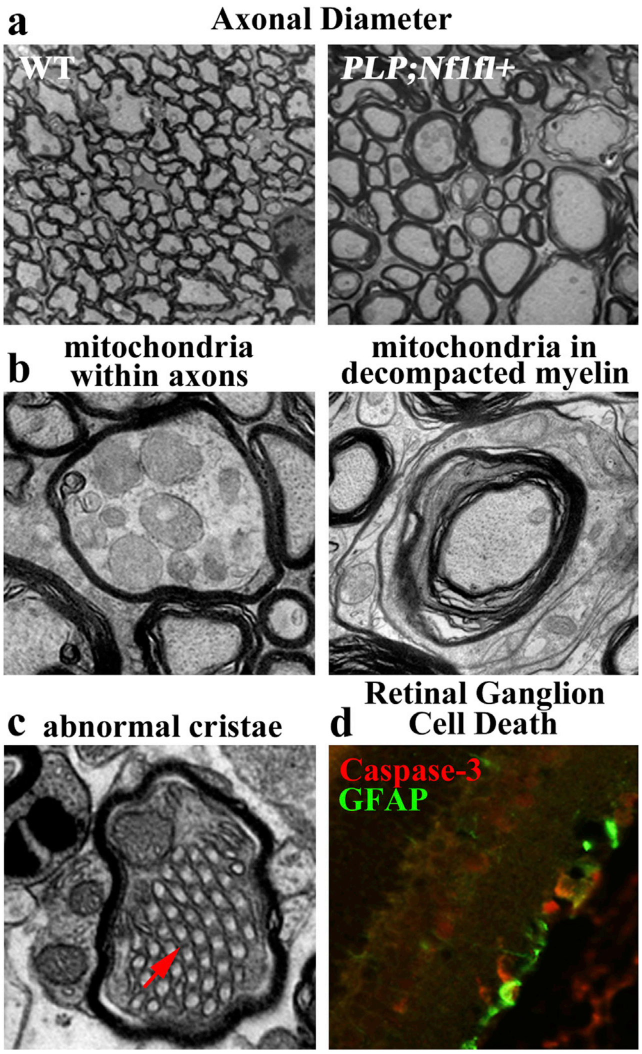

Figure S4. Increased Axon Diameter Correlates with Alterations in Mitochondria and Neuron Death, Related to Figure 1
(A–C) Electron micrographs of optic nerve crossections: (A) WT and *PLP;Nf1fl/+* (10,000x), (B) *PLP;Nf1fl/fl* (20,000X), and (C) *CNP-HRas* (50,000X) with abnormal mitochondrial cristae = red arrow.
(D) Immunohistochemistry of *PLP;Nf1fl/fl* retina double-labeled with Caspase-3 and GFAP.

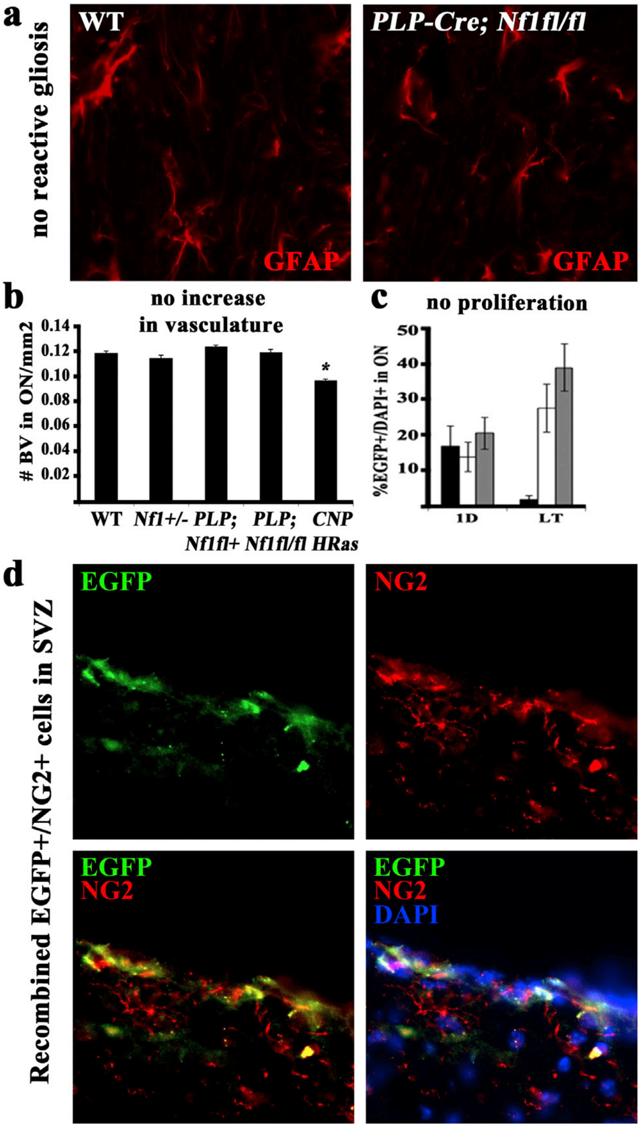

Figure S5. Optic Nerve Enlargements Are Not a Result of Optic Glioma Formation, Related to Figure 3

(A) GFAP immunohistochemistry in the optic nerves of wild-type (WT) and *PLPCre;Nf1fl/fl* animals four weeks post adult tamoxifen injection.

(B) Graph of the number of blood vessels (BV) per mm^2 measured from optic nerve semithin crossections 1mm rostral to the chiasm (n = 5/genotype).

(C) Graph of the % of EGFP+ cells to total DAPI+ cells in optic nerve crossections either 1 day or 12 months post adult Tamoxifen in *PLPCre;Nf1fl/fl* mice (n = 3/time point).

(D) Immunohistochemistry showing double-labeled EGFP+/NG2+ progenitor cells in the subventricular zone (SVZ).

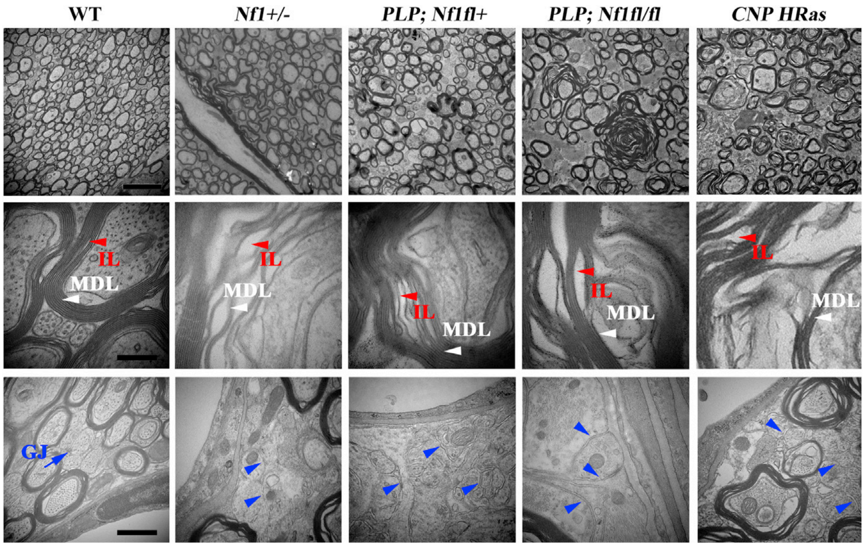

Figure S6. Myelin Decompaction and Perivascular Changes Are Mimicked in the Corpus Callosum after *Nf1* Loss of HRas Activation, Related to Figure 4

Electron micrographs of corpus callosum (top = 10,000X, scale bar = 2 μm; middle = 50,000X, scale bar = 100nm; lower = 20,000X, scale bar = 1 μm). White arrowhead = major dense lines (MDL). Red arrowhead = intraperiod lines (IL). Blue arrows = gap junctions on astrocyte endfeet (GJ). Blue arrowheads = astrocyte endfeet without gap junctions. n = 3-5 animals/genotype.

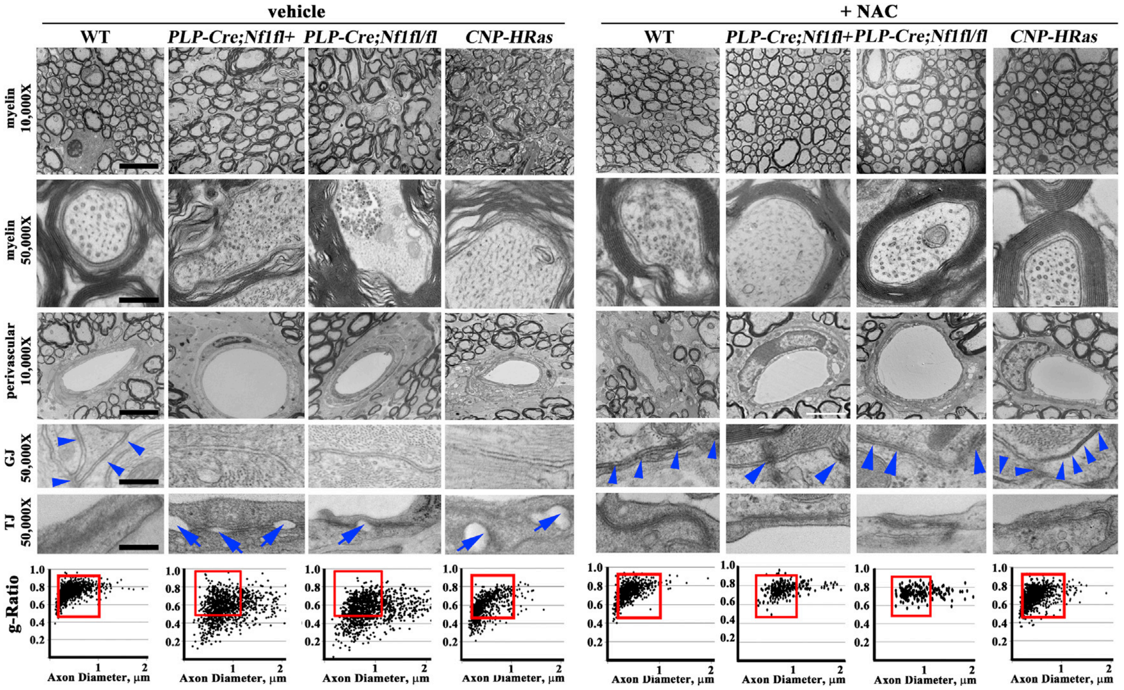

Figure S7. Rescue of Myelin Compaction, Perivascular Size, and Gap and Tight Junction Expression after NAC Administration to *PLPCre;Nf1fl/+; PLPCre;Nf1fl/fl*, and *CNP-HRas* Animals, Related to Figure 4

Electron micrographs of wild-type (WT), *PLPCre;Nf1fl/+, PLPCre;Nf1fl/fl*, and *CNP-HRasG12V* (CNP-HRas) animals after 6 weeks of administration of either vehicle or NAC treatment. Optic nerve crossections 1mm rostral from the chiasm at 10,000X (scale bar = 2 µm), and 50,000X (scale bar = 100nm). Electron micrographs crossections of capillaries and perivascular space in optic nerve 1mm from chiasm (10,000X). Electron micrographs of astrocyte endfeet surrounding optic nerve capillaries (50,000X). Blue arrowheads = Gap junctions (GJ) on astrocyte endfeet. Electron micrographs show endothelial cell tight junctions (50,000X). Blue arrows = tight junction gaps. Scatter plots of g-Ratio raw data (>1,000 axons/graph) of optic nerve measured from the pre-chiasm 1mm rostral from the chiasm of 3-5 electron micrographs (10,000X) of 3-5 animals per genotype. Red box = 99% of wild-type (WT) g-Ratios superimposed upon graphs of other genotypes.
